# Mechanical stress alters protein O‐GlcNAc in human periodontal ligament cells

**DOI:** 10.1111/jcmm.14509

**Published:** 2019-06-25

**Authors:** Dorottya Frank, Annamária Cser, Béla Kolarovszki, Nelli Farkas, Attila Miseta, Tamás Nagy

**Affiliations:** ^1^ Department of Dentistry, Oral and Maxillofacial Surgery, Medical School University of Pécs Pécs Hungary; ^2^ Institute of Bioanalysis, Medical School University of Pécs Pécs Hungary; ^3^ Department of Laboratory Medicine, Medical School University of Pécs Pécs Hungary

**Keywords:** O‐GlcNAc, orthodontic tooth movement, Periodontal ligament cells, post‐translational modifications, stress response

## Abstract

Protein O‐linked N‐acetylglucosamine (O‐GlcNAc) is a post‐translational modification of intracellular proteins that regulates several physiological and pathophysiological process, including response to various stressors. However, O‐GlcNAc's response to mechanical stress has not been investigated yet. As human periodontal ligament (PDL) cells are stimulated by compression force during orthodontic tooth movement that results in structural remodelling, in this study we investigated whether mechanical stress induces any alteration in protein O‐GlcNAc in PDL cells. In this study, PDL cells isolated from premolars extracted for orthodontic indications were exposed to 0, 1.5, 3, 7 and 14 g/cm^2^ compression forces for 12 hours. Cell viability was measured by flow cytometry, and protein O‐GlcNAc was analysed by Western blot. Cellular structure and intracellular distribution of O‐GlcNAc was studied by immunofluorescence microscopy. We found that between 1.5 and 3 g/cm^2^ mechanical compression, O‐GlcNAc significantly elevated; however, at higher forces O‐GlcNAc level was not increased. We also found that intracellular localization of O‐GlcNAc proteins became more centralized under 2 g/cm^2^ compression force. Our results suggest that structural changes stimulated by compression forces have a significant effect on the regulation of O‐GlcNAc; thus, it might play a role in the mechanical stress adaptation of PDL cells.

## 1 INTRODUCTION

Orthodontic mechanotherapy is based on the principle that prolonged pressure applied to the teeth will remodel the surrounding bone and periodontal structures and ultimately enable orthodontic tooth movement.^1^ This remodelling process is mediated by the periodontal ligament (PDL). The mechanical stimulus induces bone resorption and absorption in compression and tension sites of the PDL, respectively. Therefore, the orthodontic tooth movement is thought to be primarily a periodontal ligament phenomenon. Despite many studies providing some insight into the biological basis of tooth movement, the complex mechanism that underlies the phenomenon is still not fully understood.^1^, ^2^

In 1984, GW Hart described a novel post‐translational protein modification called O‐linked N‐acetylglucosamine (O‐GlcNAc); a single N‐acetylglucosamine saccharide is attached to serine and threonine hydroxyl groups of nuclear and cytoplasmic proteins.^3^ Hundreds of proteins have been identified as being subjected to protein O‐GlcNAc including transcription factors, cytoskeletal and signalling components and metabolic enzymes.^4^ This modification is highly dynamic when responding to various stimuli. Protein O‐GlcNAc shares some features when compared to serine/threonine phosphorylation, such as the existence of cyclic enzymes or the occupation of the same residues on proteins.^5^ There is compelling evidence that O‐GlcNAc is involved in the regulation of several physiological (transcription,^6^ nutrient sensing 7 and cell cycle regulation 8) and pathological processes (diabetes,^9^ cancer,^10^ Alzheimer disease 11). For example, in patients with diabetes, chronic hyperglycaemia will lead to elevated levels of protein O‐GlcNAc modification 12 which could have several deleterious consequences, such as altered transcriptional factor activity or interference with phosphorylation.^13^ Importantly, recent researches have also found altered O‐GlcNAc levels associated with increased stress tolerance. In conditions such as oxidative stress, hypoxia or heat shock, the regulatory role of O‐GlcNAc in cell survival and adaptation has been proposed.^14^

Although cellular structural/cytoskeletal elements responsible for cellular motility and the dynamic maintenance of cellular shape such as actin, vimentin and tubulin are all influenced by O‐GlcNAc modification,^15^, ^16^ the impact of mechanical stress on protein O‐GlcNAc has not been investigated yet. Given the large number of proteins modified by O‐GlcNAc, its regulation by mechanical stress would also impact the transcriptional activity, proliferation rate, motility and resistance to deformation of PDL cells. Moreover, analysis of O‐GlcNAc in PDL cells would help to predict disorders of orthodontic tooth movement in pathologic conditions such as diabetes.

In our present study, we have been suggested that mechanical compression may have an effect on O‐GlcNAc levels in PDL cells and, accordingly, protein O‐GlcNAc modification might play role in mechanical stress adaptation. Therefore, we investigated the effect of mechanical load on the level of protein O‐GlcNAc in human PDL cells in vitro, in order to better understand the biological basis of orthodontic tooth movement.

## 2 MATERIALS AND METHODS

### 2.1 Cells and culture conditions

Primary human PDL cells were isolated from healthy, non‐carious first premolars undergoing tooth extraction for orthodontic indications according to the method of Somerman et al 17 with slight modifications. Briefly, periodontal tissues were collected with a scalpel from the central third of the roots. Tissues were further cut into smaller (~ 0.1‐0.5 mm^3^) pieces to allow PDL cells to migrate out from the connective tissue and occupy the surface of the tissue culture flask as a confluent monolayer. The morphology of the PDL cell cultures confirmed mainly fibroblast‐like cells, spindle‐form cells, large cytoplasm‐to‐nucleus ratio, single cells capable to migrate and forming large bundles, without any apparent contact inhibition (Appendix Figure A1). The cells were cultured in a 1:1 mixture of EMEM and Ham's F12 medium (Lonza) supplemented with 10% foetal bovine serum (Thermo Fischer Scientific), 1% non‐essential amino acids, penicillin (100 U/mL, Sigma‐Aldrich^™^) and streptomycin (100 µg/mL, Sigma‐Aldrich^™^). The cells were incubated at 37°C, 5% CO2 in a humidified incubator. Subculturing was performed after reaching confluency. The medium was refreshed 12‐24 hours prior to each experiment. At least 5 independent human PDL primary cell lines were obtained, and experiments were carried out between the third and fifth passages. Prior to sample collection, written informed consents were obtained from all patients. The procedures were approved by the Regional Committee for the Research Ethics (Ref.No.: 6133).

### 2.2 Application of mechanical stress

To simulate continuous mechanical stress, a previously described compression method was used.^18^, ^19^ Briefly, PDL cells were plated onto 6‐well cell culture plates (VWR International) at density of 5 × 10^4^ cells per cm^2^. After confluency reached, sterile, 30‐mm‐diameter coverslips were placed on top of the PDL cell monolayers. The cells were then subjected to 0 (just coverslip), 1.5, 3, 7 and 14 g/cm^2^ compressive force for 12 hours. Monolayer cells not covered with coverslip served as control. After mechanical stress application, the cells were harvested and subjected to viability testing by flow cytometry and Western blot analysis as described below.

### 2.3 Viability assay

Cells were detached from the cell culture plates by incubation in phosphate‐buffered saline (PBS) containing 0.25% trypsin (Sigma) and 0.5 mmol/L EDTA at 37°C. Resuspended cells were washed in complete medium to neutralize trypsin and washed in ice‐cold PBS. Next, the cells were stained with propidium iodide (PI) according to the manufacturer's protocol (BD Pharmingen, Cat. No.: 556420). The fluorescence intensity of PI dye per cell was detected at 620 nm (FL3 channel) with Cytomics FC 500 flow cytometer (Beckman Coulter). Parallel with the fluorescence intensity detection, forward light scatter (FS) values were detected as a function of cell size. Defining the region of live and dead cells was performed on control samples, and identical boundaries were utilized for all samples.

### 2.4 Western blot analysis

After the careful removal of weights and coverslips, the PDL cell layers were washed 2x in ice‐cold PBS and harvested in RIPA buffer [10 mmol/L Tris pH 7.2, 100 mmol/L NaCl, 1 mmol/L EDTA, 1 mmol/L EGTA, 0.1% SDS, 1% Triton‐X 100, 0.5% deoxycholate, 10% glycerol, protease inhibitor cocktail: 1 tablet/10 ml (Sigma‐Aldrich^™^)], kept on ice for 30 minutes. and centrifuged for 10 minutes. at 4°C at 14 000 × g. Following centrifugation, the supernatant was used to determine total protein concentration by using Bio‐Rad Dc Assay Kit (Bio‐Rad). Proteins were separated on 8% SDS‐PAGE and transferred onto polyvinylidene difluoride membranes (Millipore). Blots were probed by RL2, an anti‐O‐GlcNAc monoclonal mouse IgG antibody (1:1000; Thermo Fisher Scientific, Cat. No.: MA1‐072) in 5% non‐fat dry milk blocking buffer and followed by HRP conjugated goat anti‐mouse IgG (1:5000; Thermo Fisher Scientific). The blots were developed using Femto chemiluminescent substrate (Thermo Fisher Scientific), and the signal was detected by G:BOX Chemi HR1.4 gel imaging system (Syngene). As loading control, anti‐actin IgG antibody (Sigma‐Aldrich^™^, Cat. No.: A2103, 1:1500) was used. Densitometry was quantified by using ImageJ version 1.52 analysis software (National Institutes of Health, Bethesda).

### 2.5 Immunofluorescence microscopy

Cells were grown on coverslips in 6‐well plates until approx. 15% confluency in complete media. Subsequently, the coverslips were turned over; thus, the cellular layers were put between the coverslips and the surface of the plates. The coverslips were either subjected to 0 (just coverslip) or 2 g/cm^2^ compressive force for 12 hours. Control cells were kept on the coverslips in upside position. At the end of the treatments, all coverslips were turned back upside carefully, the media was removed, and the cells were washed with ice‐cold PBS. Next, the cells were fixed in 4% PBS‐buffered paraformaldehyde for 30 minutes. at room temperature and subsequently washed with PBS. To avoid formaldehyde autofluorescence, the coverslips were quenched with 50 mmol/L ammonium chloride for 10 minutes. The cells were permeabilized with 0.25% Triton‐X 100 for 10 minutes. and then blocked with 5% bovine serum albumin in PBS for 5 minutes. Next, the coverslips were incubated at room temperature with CTD110.6 an anti‐O‐GlcNAc monoclonal antibody (Sigma‐Aldrich, Cat.No.:O7764, 1:200) for 2 hours in 5% BSA/PBS and also with either anti‐actin (Sigma‐Aldrich, Cat.No.:A2103, 1:100) or anti‐α‐tubulin (Sigma‐Aldrich, Cat.No.:T8203, 1:100) or anti‐vimentin (Thermo Fisher Scientific, Cat.No.:MA5‐16409, 1:100). The samples were incubated with the secondary antibody for 2 hours. For CTD110.6, Alexa Fluor 594 goat anti‐mouse IgM secondary antibody (Life Technologies, 1:200), for tubulin, FITC goat anti‐mouse IgG secondary antibody (Thermo Fisher Scientific, 1:100) and for actin and vimentin, FITC goat anti‐rabbit IgG secondary antibody (Thermo Fisher Scientific, 1:100) were used. Nuclei were counterstained with Hoechst dye at a concentration of 1 µg/mL for 15 minutes. at room temperature. Finally, coverslips were mounted with Vectashield (Vector Laboratories) mounting medium. Image acquisition was performed with a Nikon Eclipse Ti‐U (Nikon Instruments Europe BV) inverted fluorescent microscope with NIS‐Elements Br 4.40 Imaging software (Nikon Instruments Europe BV). ImageJ software was used to calculate the average distance of O‐GlcNAc‐positive regions from the nuclei, employing binary transformation and the “Analyze particle” function of the software (Appendix Figure A2).

### 2.6 Data analysis

Data are presented as mean ± standard deviations (SD) throughout. Comparisons were performed by one‐way ANOVA plus Dunnett's post hoc test using SPSS software. Statistically significant differences between groups were defined as *P* Values < 0.05 and are indicated in the figure legends.

## 3 RESULTS

### 3.1 Cell viability

**Figure 1:**
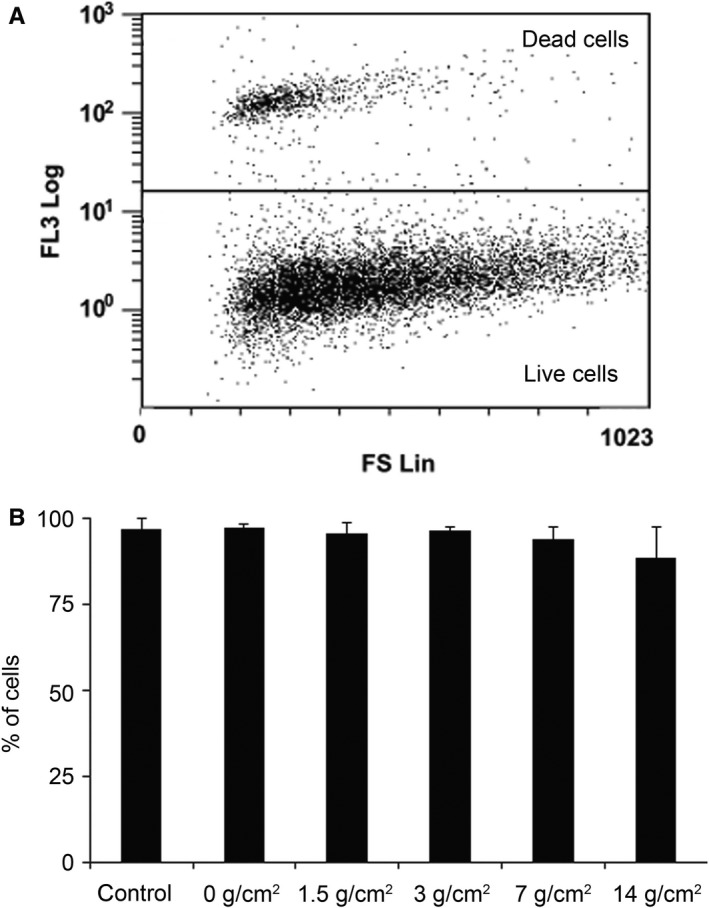
Periodontal ligament cells survive mechanical compression for 12 h. Cells were exposed to 0‐14 g/cm^2^ of mechanical compression for 12 h. The ratio of living cells was measured using propidium iodide (PI) viability assay via flow cytometry. (A) Representative dot plot chart of PDL cells compressed by 7 g/cm^2^ force for 12 h. PI staining (y‐axis, FL3 Log) was plotted against forward scatter (x‐axis, FS Lin) which is proportional to cell size. Live (bottom region) and dead (upper region) cells were separated based on FL3 signal intensity (PI staining). (B) Average ratio of live cells compared with the total number of cells. Bars are representing mean values ± SD from at least 3 independent experiments. *P < 0.05 vs control

We used compression force of up to 14 g/cm^2^ to test the mechanical resistance of cultured PDL cells. Control samples were incubated for the same time period but did not receive any mechanical load. Cells in the 0 g/cm^2^ group were covered with only a coverslip. The average mechanical load because of the coverslips on the cells is ~26 ± 0.4 mg, that is <2% of the next, smallest load in our experimental setup. We assessed the cell viability after 12 hours of mechanical compression by PI staining. As shown in Figure 1A, dead cells could be clearly separated from live cells by their increased PI uptake. The viability of control cells was 97.1 ± 2.88%, whereas the viability of the 0 g/cm^2^ group was similar, 97.5 ± 1.1%. The percentage of living cells in the compressed groups were not significantly different from either the control or the 0 g/cm^2^ group (*P* = 0.390); however, we found a decreasing tendency towards the increased mechanical compression. Viability was 95.6 ± 3.29% in the 1.5 g/cm^2^ group, which decreased to 88.8 ± 8.6% in the 14 g/cm^2^ group (Figure 1B).

### 3.2 Mechanical stress altered protein O‐GlcNAc

**Figure 2:**
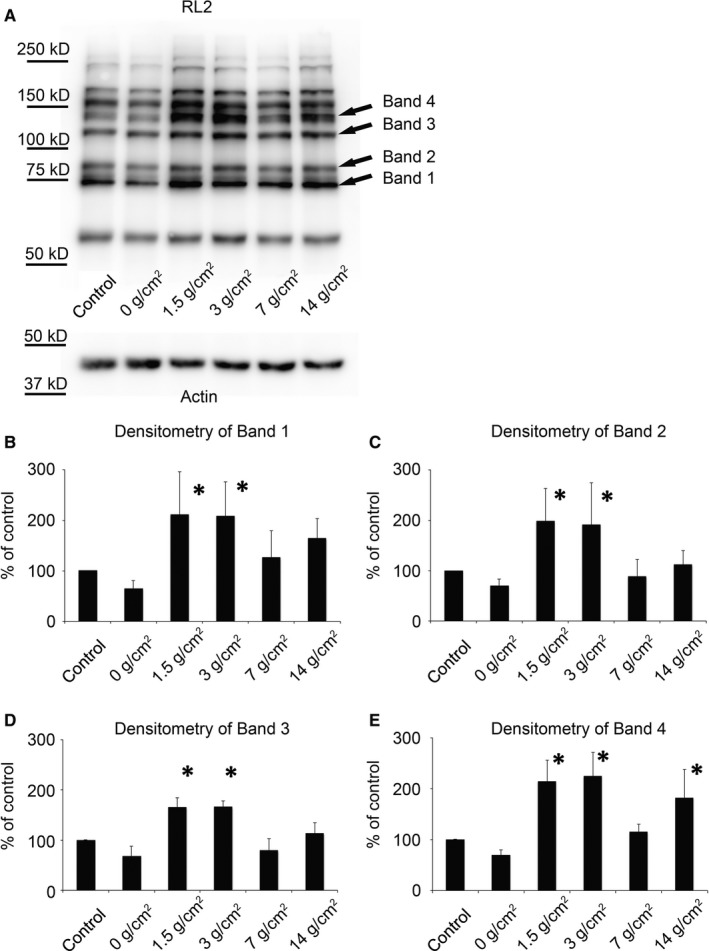
Mechanical stress alters protein O‐GlcNAc in human PDL cells. PDL cells were subjected to 0, 1.5, 3, 7 and 14 g/cm^2^ compression force for 12 h. (A) Representative Western blot of total cellular extracts. Proteins were separated by 8% SDS‐PAGE, and levels of O‐GlcNAc were determined by RL2, an anti‐O‐GlcNAc antibody. Immunoblots for actin were used as loading control. (B‐E) Densitometry was performed on immunoblots normalized to actin. 4 bands indicated by arrows in panel (A) were selected for densitometric analysis. Protein O‐GlcNAc levels are expressed as a percentage of control samples. Data are mean values ± SD from at least 5 independent experiments. *P < 0.05 vs control

The effect of mechanical stress on O‐GlcNAc levels was investigated by immunoblotting of PDL cell extracts. In response to 12 hours mechanical compression, several protein bands showed significant changes in O‐GlcNAc that depended on the magnitude of the mechanical compression (Figure 2A‐E and Appendix Figure A3). The densitometric analysis of 3 selected bands (at ~70, 85, 115 and 140 kD) is summarized in Figure 2B‐E (indicated by the arrows in Figure 1A, Band 1‐4, respectively). When only coverslips were used, protein O‐GlcNAc levels showed a slight (~30%) decrease at every selected bands. In contrast, 1.5 g/cm^2^ force resulted in significant, approximately ∼2‐fold increase at every bands. The smallest increase was seen at Band 3 (165.8 ± 18.6%) (*P* < 0.01), while the highest at Band 4, where O‐GlcNAc level increased up to 215.5 ± 41.1% (*P* < 0.01). Similarly, 3 g/cm^2^ compression doubled O‐GlcNAc levels at every selected bands, for example at Band 4 it increased significantly (*P* < 0.01) up to 225.72 ± 46.48%. On the other hand, at 7 g/cm^2^ mechanical compression the staining intensities were similar to those samples where no forces were applied (control). However, when the greatest, 14 g/cm^2^ force were used, O‐GlcNAc levels showed again a modest increase, which was only significant at Band 4 (*P* = 0.014) when compared to the control.

### 3.3 Immunofluorescence detection shows altered O‐GlcNAc distribution in compressed PDL cells

**Figure 3:**
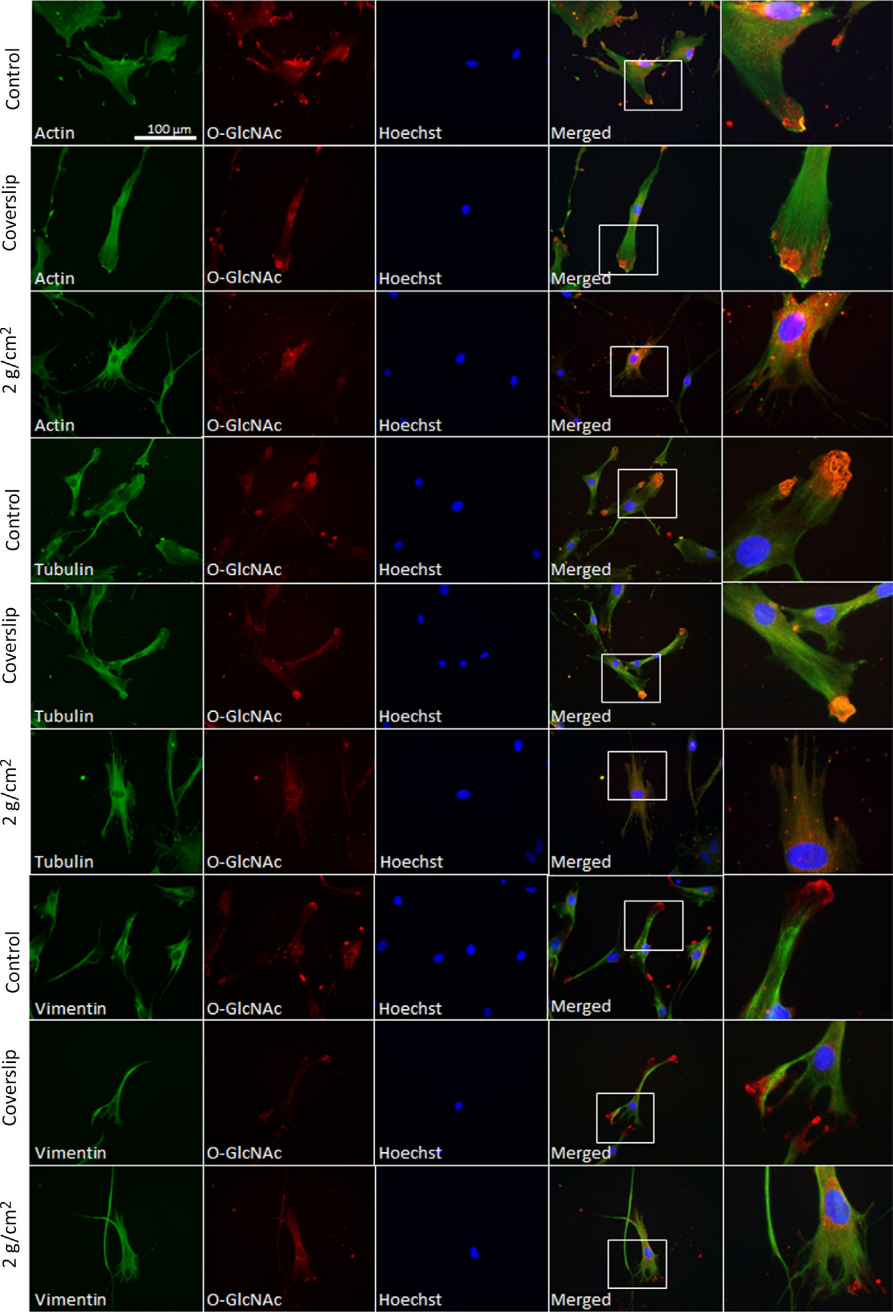
Protein O‐GlcNAc distribution pattern compared with actin, tubulin and vimentin staining after compression stress in human PDL cells. PDL cells were grown on coverslips (control), or the coverslips were turned upside‐down for 12 h (coverslip) or turned upside‐down and compressed with 2 g/cm^2^ force for 12 h (2 g/cm^2^). Immunofluorescence labelling was performed on PDL cells fixed on coverslips. Representative epi‐fluorescence images of cells stained with anti‐cytoskeletal filament antibodies (actin, tubulin or vimentin—green, first column), anti‐O‐GlcNAc antibody CTD110.6 (red, second column) and Hoechst nuclear staining (blue, 3rd column) are shown. Corresponding merged images are shown in the 4th column, whereas higher magnification images (outlined by squares in the merged images) displaying membrane protrusions are shown in the 5th column

We have also investigated the relationship between the cytoskeletal filaments and O‐GlcNAc modification after compression stress. Thus, we simultaneously labelled PDL cells with CTD110.6 and either anti‐actin, anti‐tubulin or anti‐vimentin antibodies. O‐GlcNAc staining was granular and relatively abundantly distributed in the cytoplasm. Interestingly, O‐GlcNAc was specifically enriched in membrane protrusions in control, untreated cells and in cells pressed with coverslip only. In cells exposed to 2 g/cm^2^ compression force, these membrane protrusions seemed to be retracted and narrowed. Correspondingly, O‐GlcNAc staining also tended to be more centralized (Figure 3). According to our calculations, the average distance of O‐GlcNAc stained regions from the nuclei significantly decreased in compressed cells (19.18 ± 11.2 µm vs 31.8 ± 23.7 µm) when compared to control cells (Table 1.). Albeit elevated, intracellular distribution of O‐GlcNAc modified proteins did not change in cells when O‐GlcNAc formation was only chemically stimulated (Appendix Figure A4).

**Table 1:** Average distances of O‐GlcNAc‐positive regions from the nuclei

	Distance from the nuclei	Average cell size	Distance × 100/cell size
control	31.8 ± 23.7 µm	4970 ± 4950 µm^2^	0.75 ± 0.30
coverslip	23.9 ± 15.1 µm	4570 ± 1910 µm^2^	0.68 ± 0.68^#^
2 g/cm^2^	19.18 ± 11.2 µm	4852 ± 2189 µm^2^	0.43 ± 0.27^‡^

Note

Data are means ± SD, n = 12 cells/ group

^#^ *P* = 0.763 vs control

^‡^ *P* = 0.016 vs control.

Actin, tubulin and vimentin showed fibrillar morphology and, in general, little co‐localization with O‐GlcNAc. Actin and tubulin filaments also participated in the formation of the lamellipodial‐shaped membrane protrusions, whereas vimentin was not detected at the edge of the cells. Interestingly, tubulin and O‐GlcNAc labelling seemed to be overlapping in these membrane protrusions. To a lesser extent, actin and O‐GlcNAc overlap was also noticeable in these locations; however, vimentin (because of its apparent absence in the protrusions) showed no sign of co‐localization in any of the experimental conditions. Following compression stress, both actin and tubulin staining showed that lamellar morphology changed to filamental morphology at the periphery of the cells. On the other hand, there was no noticeable change in vimentin staining.

## 4 DISCUSSION

In this study, we demonstrated for the first time that protein O‐GlcNAc is significantly altered in response to mechanical compression in human PDL cells in vitro. PDL cells enduring 1.5‐3 g/cm^2^ pressure for 12 hours developed a significant increase in O‐GlcNAc levels compared with control. The intracellular distribution of O‐GlcNAc‐rich proteins also changed upon compression force. Our results suggest that the regulation of O‐GlcNAc has an “optimal” range in relation to mechanical challenges.

The orthodontic force is an extrinsic mechanical stimulus that evokes cellular responses and therefore allows orthodontic tooth movement.^20^, ^21^ The biologic effect also depends on the PDL area over which the force is distributed; therefore, the net effect should be considered as force per unit area or pressure. Ren et al have concluded that very small pressure (<2 g/cm^2^) can stimulate biologic responses. However, neither the exact threshold nor the optimal pressure magnitude could be defined.^22^ Other studies found that light force is preferable to avoid potential overloading that can hinder tooth movement.^23^ In most in vitro studies, compression force was used in the range of 0‐4 g/cm^2^ and 2‐3 g/cm^2^ was found to induce the most significant changes.^18^, ^19^, ^24^ Our results correlate with these studies as O‐GlcNAc changes were most prominent at 1.5‐3 g/cm^2^ compression force. In contrast to in vitro studies, large individual differences have been shown in response to orthodontic forces in vivo. Early animal studies have suggested that a continuous forces of not more than 15‐20 g/cm^2^ should be used for optimum biological tooth movement, as higher forces may lead to adverse effects.^25^ More recently, forces between 1.2 and 10 g for up to 14 days were used to move rat molars.^26^, ^27^ This discrepancy from in vitro findings could be explained by different bone/mineral density, variation in individual anatomic structures, or different structure of the collagen fibres and cellular activity. One of the limitations of our study is that we have used beta‐actin as an internal control for the Western blot analyses. Actin is widely used as a general loading control in stress‐related Western blot studies 28, 29; on the other hand, its dynamic nature is a key player in the cellular motility and morphology. Nevertheless, a few studies suggest that the overall level of actin is not changing significantly after mechanical stress.^19^, ^30^

The success of orthodontic tooth movement depends largely on the remodelling capability of alveolar bone. Impaired glucose metabolism in diabetes has been demonstrated to influence bone metabolism and bone formation.^31^, ^32^ Several studies have reported significant differences in bone response to orthodontic stimulus under diabetic conditions.^31^, ^33^ Furthermore, metformin administration was found to diminish the adverse effects of diabetes on tooth movement.^31^ There is an increasing number of evidences demonstrating a strong linkage between increased O‐GlcNAc and diabetic complications.^34^ Our results indicating that O‐GlcNAc regulation is part of the PDL's response to mechanical stress might provide an important insight into the molecular mechanisms associated with altered biologic response during orthodontic tooth movement of patients with diabetes. Chronic imbalance of O‐GlcNAc because of hyperglycaemia might lead to several unwanted effects,^13^ possibly also interfering with orthodontic tooth movement.

In contrast to diabetes, in acute stress events, increased O‐GlcNAc modification is thought to be mainly beneficial. Several studies found that a wide variety of stressors, including hypoxia, hypoglycaemia and heat shock in various tissues elevates O‐GlcNAc levels.^14^ Although metabolic and chemical stimuli were extensively used to elicit changes in O‐GlcNAc, no studies were carried out to test mechanical challenges. Here we have shown for the first time that PDL cells exposed to mild mechanical stimuli increased O‐GlcNAc levels. Moreover, we have found that O‐GlcNAc is enriched at lamellipodia‐like peripheral membrane protrusions under normal, non‐compressed condition which mostly overlapped with tubulin filaments. Compression force disrupted this morphology and resulted in the retraction of cytoskeletal structure and O‐GlcNAc proteins as well. Cellular motility and lamellipodia formation is a complicated, dynamical process still only partially understood.^35^ Apart from physical forces such as membrane tension, traction forces or intracellular pressure,^36^ external chemotaxis and intracellular signalling events also influence membrane protrusions.^35^, ^37^ Actin is playing a major part in the dynamic formation of the leading edge, whereas microtubules also seem to play an important part in motility.^38^, ^39^ In our experiments, O‐GlcNAc seemed to overlap mostly with microtubules. Although tubulin was demonstrated to be O‐GlcNAc modified,^40^ we think that the similar location of tubulin and O‐GlcNAc in membrane protrusions is 2 different consequences of the same force rather than a direct co‐localization event. Nevertheless, O‐GlcNAc regulation likely will join other signalling events 41 activated by external physical forces.

The exact role of O‐GlcNAc modification in orthodontic tooth movement is yet to be found. Nagel et al showed that protein needed for bone formation required O‐GlcNAc modification.^42^ PDL may consist of fibroblasts, endo‐ and epithelial cells, osteoblasts and stem cells. However, fibroblast‐like cells are predominant in the cell cultures derived from PDL.^43^ The regulation of O‐GlcNAc in osteoblasts was investigated in a few studies, showing that osteoblast differentiation is influenced by O‐GlcNAc.^42^, ^44^ O‐GlcNAc has been also attributed to hypertrophic differentiation in chondrocytes.^45^ Most importantly, a recent paper by Gu et al demonstrated that PDL cells along with C2C12 myoblast cells are influenced by O‐GlcNAc modification.^46^ Interestingly, in contrast to the study of Koyama et al,^44^ in which they found that O‐GlcNAc promotes osteoblast differentiation in mouse MC3T3‐E1 cells, Gu et al found that it inhibited osteoblast differentiation. Despite this controversy, it seems to be that O‐GlcNAc may have a crucial role in the regulation of PDL cells. In particular, stress adaptation mechanisms by O‐GlcNAc might be an important function in PDL cells. In our experiments, we have seen no significant decrease in cell viability even at the highest load; however, it is possible that longer exposure or increased weight load would lead to damage that is no longer possible to overcome. A potential limitation of our study is that hypoxia and inadequate nutrient availability (because of altered media diffusion) might have an additional effect. To control these variables, we covered the PDL cells with only coverslips. We found that in these samples protein O‐GlcNAc slightly decreased; however, this change was not significant. Therefore, all the alterations were possibly the result of the mechanical challenge and not other processes.

In summary, we demonstrated for the first time that mechanical stress has a significant effect on protein O‐GlcNAc levels in PDL cells. According to our results, O‐GlcNAc might play an important role in the regulation of orthodontic tooth movement. Moreover, mechanical compression and tension forces are present almost everywhere in the body, for example in the joints and bones, in the heart and lungs, and blood cells undergo shear stress in the blood. Pathophysiologic processes such as tumour growth and inflammation in closed compartments will also introduce mechanical challenges to cells. Thus, we think that our data may contribute to the better understanding of these processes.

## CONFLICT OF INTEREST

The authors confirm that there are no conflicts of interest.

## AUTHOR CONTRIBUTION

DF, AC, BK and TN performed the research; TN and AM designed the research study; BK contributed essential reagents or tools; NF, DF and AM analysed the data; and DF and TN wrote the paper.
